# Consultations by Asylum Seekers: Recent Trends in the Emergency Department of a Swiss University Hospital

**DOI:** 10.1371/journal.pone.0155423

**Published:** 2016-05-18

**Authors:** Martin Müller, Karsten Klingberg, David Srivastava, Aristomenis K. Exadaktylos

**Affiliations:** Emergency Department, Bern University Hospital, Bern, Switzerland; National Institutes of Health, UNITED STATES

## Abstract

**Background:**

Large-scale war-related migration to Switzerland and other European countries is currently challenging European health systems. Little is known about recent patterns and trends in Emergency Department (ED) consultations by Asylum Seekers (AS).

**Methods:**

A retrospective single-centre analysis was performed of the data from all adult patients with the official status of “Asylum Seeker” or “Refugee” who consulted the ED of Bern University Hospital, Switzerland, between June 2012 and June 2015. Patient characteristics and clinical information, such as triage category, type of referral and discharge, violence-related injury and diagnostic group on discharge, were extracted from the computerised database or determined from the medical reports. Changes in categorical variables between the three studied years were described.

**Results:**

A total of 1,653 eligible adult patients were identified in the 3-year period. Between the first (06/12–06/13) and third periods (06/14–06/15), the number of presentations per year increased by about 45%. The AS came from 62 different nations, the most common countries being Eritrea (13%), Somalia (13%) and Syria (11%). The mean age was 33.3 years (SD 12.3) and two thirds (65.7%) were male. The proportion of women increased over time. Moreover the relative proportions shifted from patients between 20 and 50 years to patients of under 20 or over 60 years. Nearly two thirds of the patients were walk-in emergencies and this proportion increased over time. The mean triage score was 2.9 (SD 0.7), with more than 90% presenting as “urgent consultation”. About half of the patients were treated for trauma (17.2%), infections (16.8%) or psychiatric problems (14.2%). Trauma was seen in a higher proportion of male than female patients. About 25% of the patients were admitted for in-hospital treatment.

**Conclusions:**

The recent rise in AS in the population has lead to an increase in AS presenting to EDs. This changes the composition of ED patients and should raise awareness that changes in procedures may be needed. Infectious diseases and psychiatric problems remain a heavy burden for AS presenting in the ED. A trend towards an increasing proportion of walk-in patients to the ED could not be explained by this study. Further studies and surveillance are needed to investigate this trend.

## Background

Political changes in the Middle East and Africa, with ongoing conflicts in Syria and Iraq, have led to an enormous increase in AS in Europe and Switzerland. According to the Swiss Federal Agency of Immigration, in 2014 there was a 10.7% increase in AS in Switzerland and a 35% increase in Europe. The latest statistics for 2015 show a striking increase of 66% in registered AS in Switzerland and a two-fold increase in Europe [1–3].

Surveys and registry based studies from several European countries have reported disparate results in the utilisation of emergency health care services by AS [4–6]. An overall increase in ED visits by AS has been described as the result of cultural differences, poor knowledge of local health care systems, and language barriers [7–9].

A previous study from our ED had the specific goal of identifying patients from Syria and the Middle East and gave a partial view of the new challenges in providing care for AS [10].

The ED of the University Hospital Bern, Switzerland, has a catchment area of about two million habitants and treats about 40,000 patients per year. In the last three years, there has been an increase in the number of AS and this poses a challenge for the present and near future.

In this retrospective study, we analysed the characteristics of patients with official resident status as “Asylum Seeker” or “Refugee” within a 3-year period. Assessing these new patterns and displaying trends will be important in preparing EDs for this change in practice.

## Methods

### Setting

Our Level 1 adult ED is set in the government-funded Bern University Hospital (Inselspital) in Switzerland and provides free care for every patient with or without health insurance.

### Study design and data collection

In a retrospective single-centre analysis of data between June 2012 and June 2015, all patients older than 16 with the official status of “asylum seeker” or “refugee” were identified who consulted the ED of Bern University Switzerland. The residence status and the country of origin are routinely assessed by the hospital administration and recorded in the hospital information system (SAP). If either particular was missing or the residence status was not consistent with the country of origin, the patient was excluded from the analysis. Patients under 16 years were not included in the analysis as they are usually treated in the Paediatric Emergency Department.

The following particulars for eligible patients were extracted from the computerised ED software (E-Care, ED 2.1.3.0, Turnhout, Belgium) into Microsoft^®^ Excel for Mac 2011 (Microsoft Corporation, USA) after screening the administration database and anonymising the data: patient demographics included age, gender, and country of origin, date of consultation, the triage category, referral, discharge, as well as the various text fields in the medical report.

The triage system used in our ED is the Swiss Emergency Triage Scale [11]. This is similar to the Manchester Triage System [12] and based on five categories (1 highly urgent to 5 non-urgent). Triage categories 1 (life threatening problem that requires an immediate start of treatment) to 3 (acute problem, start of treatment within 30 minutes) are defined as urgent.

The region of origin was determined by using the classification of the United Nations [13].

Missing data, diagnostic groups (based on ICD-10 main categories) and violence-related ED admissions were completed and determined by studying the medical report by the authors KK and MM.

The study was performed according to Swiss law. As data were fully anonymised prior to analysis no consent was needed (Kantonale Ethikkommission Bern, Ref. No. KEK-BE: 010/2016).

### Statistics

Data analysis was performed using Stata^®^ 13.1 (StataCorp, The College Station, Texas, USA). The mean of a distribution is presented with its standard deviation (SD), median and range.

## Results

**Fig 1:**
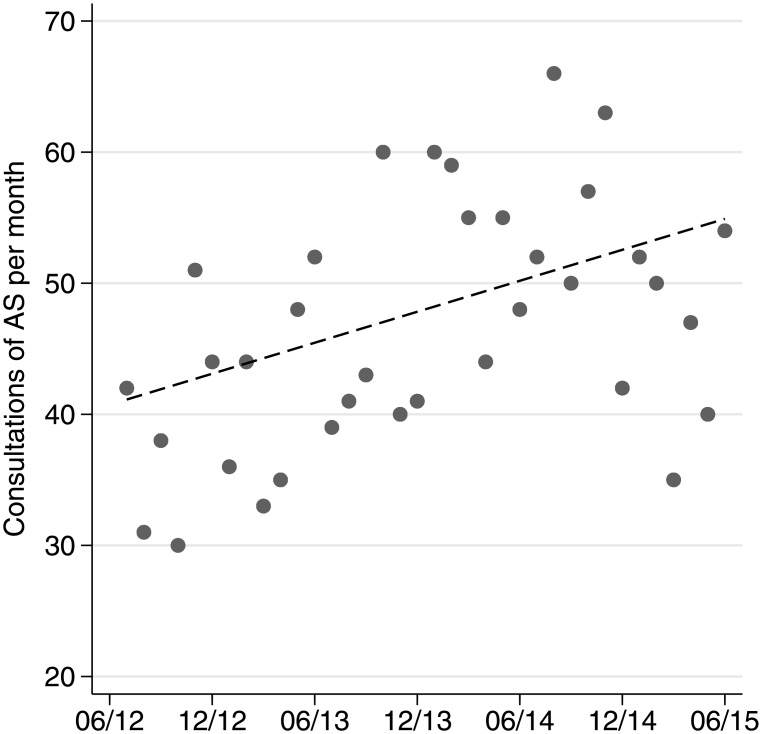
Number of consultations of asylum seekers (AS) per month from June 2015 to June 2015. A linear trend line was added to the plot.

From June 2012 to June 2015, the hospital administration system identified 1,667 patients with the residence status of AS. Fourteen patients were excluded: Four patients were excluded because the residence status was not consistent with the country of origin (United Kingdom, Spain, Switzerland). For one patient, the country was not documented, and nine patients were younger than 16 years. Thus 1,653 eligible patients were included in the analysis. Over the 3-year period, the number of patients studied per year increased from the first (06/12–06/13) to the third period (06/14–06/15) by about 45%—from 456 to 653 patients per year. The total number of patients in the ED increased in the same study period by about 33%. Fig 1 shows the number of the monthly consultations over the studied period of time.

Over half the patients originated in Eastern Africa or Western Asia (Table 1).

**Table 1:** Distribution of the region of origin, n = 1653.

Region	Freq.	Percent	Cumulative
Eastern Africa	457	27.7	27.7
Western Asia	374	22.6	50.3
Northern Africa	254	15.4	65.6
Southern Asia	218	13.2	78.8
Southern Europe	124	7.5	86.3
Western Africa	91	5.5	91.8
Eastern Europe	45	2.7	94.6
Eastern Asia	44	2.7	97.2
Middle Africa	25	1.5	98.7
Stateless	10	0.6	99.3
Northern Europe	6	0.4	99.7
South-Eastern Asia	2	0.1	99.8
Southern Africa	2	0.1	99.9
Caribbean	1	0.1	100.0
Total	1,653	100.0	

**Fig 2:**
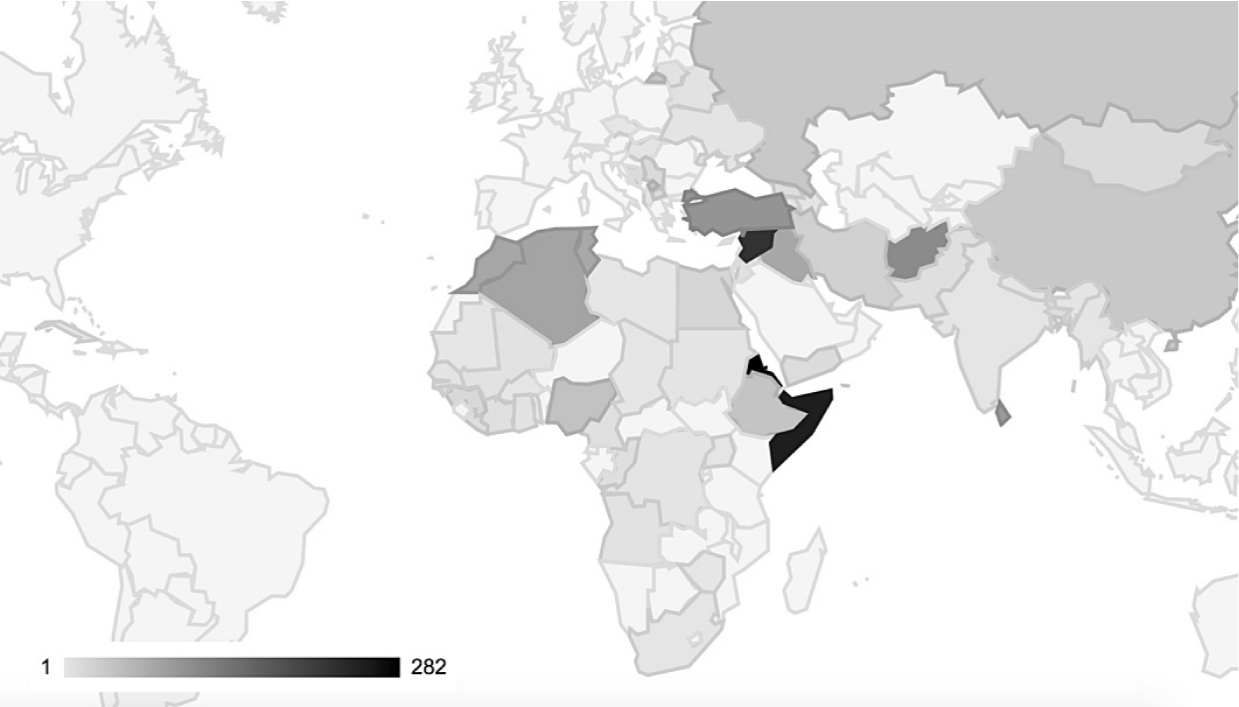
Graphical distribution of country of origin, n = 1653. The brightness of a country inversely correlates with the number of AS from this country presented at our ED between 06/13–06/15. Authors' own figure created with https://jsfiddle.net/.

The AS came from 62 different countries. The most common countries of origin were Eritrea (13%), Somalia (13%), and Syria (11%) (Fig 2). Subgroup analysis revealed no association between triage category, diagnostic group, type of admission or being an AS from Somalia rather than from another country.

The mean age was 33.3 years (SD 12.3, median 30, range 16–79), with more male (65.7%) than female patients. The proportion of male AS fell over the three year period—from 71.1% to 65.7%. There was an association between age group and the three studied periods, as both the proportion of the youngest age group (16–19) and of the oldest age group (60–79) increased over time (Table 2).

**Table 2:** Patient characteristics in each studied time period.

	6/12-6/13		6/13-6/14		6/14-6/15		Total	
**Gender, %(n)**								
Female	28.9%	(137)	33.5%	(177)	38.8%	(253)	34.3%	(567)
Male	71.1%	(336)	66.5%	(351)	61.2%	(399)	65.7%	(1,086)
Total	100.0%	(437)	100.0%	(528)	100.0%	(652)	100.0%	(1653)
**Age group, %(n)**								
16–19	7.4%	(35)	9.2%	(49)	13.8%	(90)	10.5%	(174)
20–39	68.5%	(324)	62.9%	(332)	57.4%	(374)	62.3%	(1,030)
40–59	22.2%	(105)	25.4%	(134)	22.5%	(147)	23.4%	(386)
60–79	1.9%	(9)	2.5%	(13)	6.3%	(41)	3.8%	(63)
**Countries of origin, %(n)**								
1.	Somalia 10.6% (50)		Eritrea 13.5% (71)		Somalia 15.8% (103)		Eritrea 13.1% (216)	
2.	Eritrea 10.4% (49)		Somalia 10.4%, (55)		Eritrea 14.7% (96)		Somalia 12.6% (208)	
3.	Syria 9.9% (47)		Syria 10.2% (54)		Syria 13.3% (87)		Syria 11.4% (188)	
4.	Morocco 9.7% (46)		Algeria 7.6% (40)		Afghanistan 8.1% (53)		Afghanistan 5.7% (94)	
5.	Tunisia 72% (34)		Turkey 7.0% (37)		Sri Lanka 5.1% (33)		Turkey 5.1% (84)	

Over 63% of the patients were walk-in emergencies, followed by 17% brought in by ambulance. Only 5% of the patients were referred by a general practitioner. 9% were transferred from other hospitals. The proportion of walk-in patients increased from 60.3% in 2012 to 64.3% in 2015, while the referrals from general practitioners decreased slightly, from 5.5% to 4.3%. Moreover, referral by the police decreased from 8.2% to 4.3%. Furthermore, there was an association between violence-related presentations and the studied periods: the proportion fell from 9.5% in 2012/13 to 3.5% in 2015. The mean triage score was 2.9 (SD 0.7, median 3, range 1–5), with over 90% being urgent consultations without prior appointment. There was no important difference between male or female patients. About half of the patients were treated for trauma (17.2%), infections (16.8%) or psychiatric problems (14.2%). While the proportion of trauma was higher in male patients (23.3% vs. 5.3%), the proportions of musculoskeletal (12.0% vs. 8.5%), abdominal (14.2% vs. 8.5%), and neurological problems (10% vs. 5.4%) were higher in female patients. No clear trends could be detected in the triage score and diagnostic group over the studied time period (Table 3).

**Table 3:** Distribution of type of referral, triage category, diagnostic group, and discharge in each studied time period.

	6/12-6/13		6/13-6/14		6/14-6/15		Total	
**Referral %(n)**								
Walk-In	60.3%	(285)	62.7%	(331)	64.3%	(419)	62.6%	(1,035)
Ambulance	18.8%	(89)	14.2%	(75)	17.9%	(117)	17.0%	(281)
Hospital	5.7%	(27)	11.6%	(61)	9.2%	(60)	9.0%	(148)
Police	8.2%	(39)	6.8%	(36)	4.3%	(28)	6.2%	(103)
GP	5.5%	(26)	4.2%	(22)	4.1%	(27)	4.5%	(75)
Missing	1.5%	(7)	0.6%	(3)	0.2%	(1)	0.7%	(11)
**Violence-related %(n)**								
Yes	9.5%	(45)	3.0%	(16)	3.5%	(23)	5.1%	(83)
Missing	0.9%	(4)	1.0%	(5)	0.2%	(1)	0.2%	(10)
**Triage category %(n)**								
1	5.3%	(25)	2.7%	(14)	3.5%	(23)	3.8%	(62)
2	19.7%	(93)	18.6%	(98)	21.6%	(141)	20.1%	(332)
3	63.6%	(301)	66.5%	(351)	67.5%	(440)	66.1%	(1092)
4	8.5%	(40)	7.2%	(38)	4.9%	(32)	6.7%	(110)
5	2.1%	(10)	5.1%	(27)	2.5%	(16)	3.2%	(53)
Missing	0.8%	(4)	0.0%	(0)	0.0%	(0)	0.2%	(4)
**Diagnostic group %(n)**								
Psychiatric	15.0%	(109)	13.1%	(73)	14.1%	(95)	14.0%	(277)
Musculoskeletal	8.2%	(77)	11.0%	(73)	9.4%	(106)	9.6%	(256)
Abdominal	7.6%	(70)	12.7%	(69)	11.8%	(91)	10.9%	(230)
Respiratory	3.0%	(33)	3.2%	(60)	3.1%	(75)	3.1%	(168)
Neurological	6.6%	(38)	7.2%	(57)	6.9%	(55)	6.9%	(150)
Cardiovascular	6.6%	(30)	5.5%	(38)	6.9%	(45)	6.4%	(113)
Infectious	16.3%	(31)	14.0%	(29)	16.6%	(45)	15.7%	(105)
Gynaecological	0.4%	(15)	0.8%	(33)	1.1%	(22)	0.8%	(70)
Dental	0.2%	(14)	0.6%	(17)	0.8%	(20)	0.5%	(51)
Eye	0.6%	(3)	0.9%	(3)	1.2%	(8)	1.0%	(14)
Trauma	23.0%	(2)	14.0%	(4)	14.6%	(7)	16.8%	(13)
Follow-Up	3.2%	(1)	6.3%	(3)	3.4%	(4)	4.2%	(8)
Other	8.7%	(41)	10.4%	(54)	10.3%	(65)	9.9%	(160)
Missing	0.6%	(9)	0.4%	(15)	0.0%	(14)	0.3%	(38)
**Discharge %(n)**								
Home	74.8%	(354)	73.1%	(385)	72.7%	(474)	73.4%	(1213)
Admitted	21.1%	(100)	19.4%	(102^a^)	19.8%	(129)	20.0%	(331)
Transfer	4.0%	(19)	7.2%	(38)	7.5%	(49)	6.4%	(106)
Missing	0.0%	(0)	0.4%	(2)	0.0%	(0)	0.1%	(2)

^a^One patient died in the ED.

About 75% of the patients were treated as outpatients and 6.5% were transferred to secondary hospitals. In-hospital treatment was performed in 40.0% of the patients referred by a GP and 15.2% of the walk-in emergencies.

In 2014, the overall proportion of admission (inpatient care) of all patients in our ED was 33%—ranging from 14% in young adults to 73% for very old people (90–101).

The proportion of admission in AS was found to be high at nearly 26% compared to the age-standardised (direct standardisation) overall proportion of admissions of 19% of all patients in 2014. More than two thirds (67.0%) of the transferred patients were diagnosed as having a psychiatric problem. There were no differences in type of discharge and no an association with gender.

## Discussion

Over the last two decades and particularly in the last two years, the total applications for asylum in the European Union has increased from 15,000 to over 300,000 per year [14]. This finding is caused by recent political changes and the ongoing stream of refugees seeking shelter in Europe and Switzerland [1–3].

This distinct rise in ED presentations by this population has been accompanied by new challenges related to cultural differences and language barriers [15, 16]. Problems in the treatment of AS include lack of care coverage and of knowledge of the health care system, and social deprivation [17].

This retrospective study identified a total of 1,653 AS attended the ED of Bern University Hospital, Switzerland, between June 2012 and June 2015 –an average of nearly two AS per day. The global trend for increasing ED consultations [18] is also apparent in our ED, with a relative increase of 33% over the studied period. In contrast, the number of AS presenting in our ED increased by 45%, suggesting a change in the composition of the ED patient group, and which may necessitate changes in procedures, such as additional education and training for ED staff.

Despite the ongoing conflict in Syria, a major humanitarian crisis with 2.9 million refugees [19], the leading countries of origin did not differ during the last 3 years in our ED. AS from Eritrea are the largest population of AS presenting in our ED. This is because Eritreans make up more than one quarter of all AS in the Canton of Bern [20]. Although only about 3% of all AS in the Canton of Bern are from Somalia [20], they are responsible for 16% of consultations in our ED. This finding contradicts an analysis from 2011, in which it was concluded that Somali refugees enjoyed better health than other AS populations [21]. Our subgroup analysis failed to explain this discrepancy, but we are unable to exclude bias due to frequent presentations or follow-up visits.

Over the studied period, some changes in the socio-demographic distribution were detected: Firstly, there was a shift towards more women and fewer men and, secondly, the age distribution changed; there were more patients younger than 20 years or older than 60 years, although the mean age of AS was still 33.3 (SD 12.3) years. The changes in age and gender distribution cannot be explained by changes in the general population of AS in the Canton of Bern [20, 22]. One reason for this finding might be that the efforts by the state and NGOs [23] have enhanced the health awareness of the AS, particularly for their families, resulting in better adherence to the Swiss health system. On the other hand, the total number of AS referred by a GP remained low as the proportion of walk-in patients increased. Our data cannot explain this trend, but previous studies suggested that AS might have difficulties in the use of the GP health care system [7, 24]. On the other hand, most patients were triaged to category three, which is defined as an urgent level and which is usually not seen by GPs [11]. Furthermore, the high proportion of hospital admissions confirms this triage category and is in contrast to previous studies in which lower triage categories were related to AS presenting without referral [4, 7, 25]. This may be caused by the greater incidence of severe health problems and multi morbidity in AS [26, 27], but may also be explained by selection bias, as our study population was presenting at a university hospital.

Trauma was one of the main diagnoses in this population that mainly consisted of young men. It was unclear whether this was related to work or recreation. It is notable that, over the years, police referrals and violence-related injuries have steadily decreased. This might reflect growing integration efforts and preventive measures.

Infectious diseases have a high incidence in refugees and AS and were another common diagnostic group in our ED; low vaccination and hygiene standards were described as possible causes [28, 29]. Further research is needed to identify other causes and the most important infectious diseases in AS.

The third diagnostic group was”psychiatric” and psychiatric conditions have commonly been described in these vulnerable patients [10, 30, 31]. AS have a high incidence of post traumatic stress disorder and other psychiatric diseases, such as depression, psychosocial crisis and psychosis [32]. Many AS are traumatised by violent conflicts, torture and loss, followed by uncertain and hazardous travel, unfavourable living conditions and intolerance. It is not surprising that this frequently leads to psychiatric disorders. Great efforts and close collaboration between all stakeholders caring for refugees are needed to solve this complex and important challenge. As EDs are one of the main structures in this process, training in transcultural competency and sufficient resources are paramount.

Several limitations apply to this study and the conclusions must be interpreted carefully.

The study is retrospective and the study population is biased by the selection of patients presenting to a single centre ED without sufficient data on the source AS population. Furthermore, re-admissions and follow-up admissions were not excluded and this might lead to further selection bias.

Future studies on this issue should contain more extensive demographic data and should also study the impact of cultural differences on emergency health care. This could help to identify the adaptive changes needed in ED and health education programs.

## Conclusion

The recent rise in AS in the population appears to lead to an increase of AS presenting in EDs. This changes the composition of ED patients and should raise awareness that changes may be needed.

A trend of an increasing proportion of walk-in patients and a broader spectrum of AS with respect to age and gender to the ED could not be explained by this study. Further studies and surveillance are needed to investigate this trend.

Infectious diseases and psychiatric problems remain a heavy burden for AS presenting in the ED.

## Supporting Information

S1 Dataset (XLS)
